# Multi-Compartment and Multi-Host Vector Suite for Recombinant Protein Expression and Purification

**DOI:** 10.3389/fmicb.2018.01384

**Published:** 2018-06-27

**Authors:** Claudia Ortega, Daniel Prieto, Cecilia Abreu, Pablo Oppezzo, Agustín Correa

**Affiliations:** ^1^Recombinant Protein Unit, Institut Pasteur de Montevideo, Montevideo, Uruguay; ^2^Research Laboratory on Chronic Lymphocytic Leukemia, Institut Pasteur de Montevideo, Montevideo, Uruguay; ^3^Department of Developmental Neurobiology, Instituto de Investigaciones Biológicas Clemente Estable, Montevideo, Uruguay

**Keywords:** recombinant protein, RF-cloning, protein purification, expression host, expression vectors

## Abstract

Recombinant protein expression has become an invaluable tool in basic and applied research. The accumulated knowledge in this field allowed the expression of thousands of protein targets in a soluble, pure, and homogeneous state, essential for biochemical and structural analyses. A lot of progress has been achieved in the last decades, where challenging proteins were expressed in a soluble manner after evaluating different parameters such as host, strain, and fusion partner or promoter strength, among others. In this regard, we have previously developed a vector suite that allows the evaluation of different promoters and solubility enhancer-proteins, through an easy and efficient cloning strategy. Nonetheless, the proper expression of many targets remains elusive, requiring, for example, the addition of complex post-translation modifications and/or passage through specialized compartments. In order to overcome the limitations found when working with a single subcellular localization and a single host type, we herein expanded our previously developed vector suite to include the evaluation of recombinant protein expression in different cell compartments and cell hosts. In addition, these vectors also allow the assessment of alternative purification strategies for the improvement of target protein yields.

## Introduction

Obtaining enough quantities of the target protein in a soluble, pure, and homogeneous state from its natural host is very uncommon, making the expression of the target in a recombinant form a routine practice for many academic laboratories and industry. In this regard, *Escherichia coli* has been the most widely used host for recombinant protein since its introduction in 1977 for the expression of human somatostatin (Itakura et al., 1977). This is due to its easy implementation, low cost, high yields that can be obtained and a plethora of genetic tools that are available. Despite the advances achieved in the recombinant protein field, many targets cannot be expressed in a soluble and homogeneous state, requiring the evaluation of several parameters including different solubility enhancer proteins, promoters, *E. coli* strains, among others (Correa and Oppezzo, 2011, 2015). In addition, the requirement of post-translational modifications may impose restraints for the selection of the correct expression scheme. Several approaches have been developed to allow the formation of disulfide bonds in the cytosol of *E. coli*, although for some cases it may be worthwhile to direct the expression of the target protein to the periplasmic space, which contains a specialized enzymatic system for disulfide bond formation (Berkmen, 2012). In addition, the requirement of a specific glycosylation pattern for a particular target protein can make the expression in eukaryotic hosts mandatory.

Other important issue to consider is the downstream process that can have an important effect in the quantities and quality of the final product. In this regard, several tags have been developed to facilitate the purification process. Despite the fact that the polyhistidine-tag (HisTag), has been extensively used for the purification of recombinant proteins (Walls and Loughran, 2011), protein targets that are poorly expressed in *E. coli* are usually eluted with contaminants derived from the host, requiring further purification steps and reducing final yields (Bolanos-Garcia and Davies, 2006; Magnusdottir et al., 2009). Moreover, it presented a relatively poor purification performance for extracts derived from yeast, *Drosophila*, and HeLa cells (Lichty et al., 2005). An alternative purification tag includes the *Strep*-Tag^®^II that can bind to *Strep*-Tactin^®^ affinity resins (Schmidt and Skerra, 2007). This tag was further optimized to the Twin-Strep-tag^®^, which proved to be especially useful for the purification of diluted proteins as is the case of secreted proteins (Schmidt et al., 2013). Besides, there are large proteins that have been used as purification tags like maltose-binding protein (MBP) (Pattenden and Thomas, 2008), glutathione-*S*-transferase (GST) (Smith and Johnson, 1988; Harper and Speicher, 2011), and the antibody Fc-fragment (Fisher et al., 2006; Flanagan et al., 2007).

In conclusion, several parameters may have important effects in the final yield and quality of the target protein, including promoter type, fusion protein, cell compartment, selected host, and the purification strategy. All these variables require the cloning of the target gene into several plasmids. In this regard, the utilization of an effective cloning strategy combined with the possibility of introducing the same gene into multiple vectors in a parallel manner is fundamental. In the last decades, several cloning methods were proposed where restriction-free cloning (RF-cloning) has demonstrated to be very suitable to these goals (Unger et al., 2010; Peleg and Unger, 2014).

In order to overcome the solubility problems often found in recombinant protein expression, we have previously developed a vector suite for the parallel cloning of a target gene into 12 different *E. coli* expression vectors by using RF-cloning method (Correa et al., 2014). These vectors allow the evaluation of the combined effect in protein expression of two different promoters (T5 or T7) with five solubility enhancer proteins (SUMO, Trx, DsbC, MBP, or CelD) as well as no fusion protein. Given that some targets may require the exploration of additional parameters, we herein increased the versatility of the vector suite and extended it for the evaluation of recombinant protein expression in different host cells (*E. coli*, mammalian, and *Drosophila* cells) as well as different compartments within them (cytoplasmic, periplasmic, or secreted). In this updated vector suite, we also incorporated a vector for fusion with GST mainly as an alternative purification protocol for expressions done in *E. coli* together with immobilized metal affinity chromatography (IMAC) and *Strep*-Tactin^®^ affinity resins. Eukaryotic vectors with the antibody Fc fragment or Twin-Strep-tag^®^ fusion were also developed for the purification of secreted proteins. All vectors were validated by the expression of the reporter gene green fluorescent protein (GFP). Finally as case studies, we successfully expressed in a soluble and homogeneous state two challenging human proteins corresponding to the extracellular domain of the receptor tyrosine-like kinase orphan receptor 1 (ROR1-ED) (Hojjat-Farsangi et al., 2014) and the UL16-binding protein 2 (ULBP2). Our results shows that the extended vector suite allow the easy assessment of alternative purification strategies and expression host or host compartments for successful recombinant protein expression.

## Materials and Methods

### Generation of the New Vectors

The new vectors for expression in *E. coli* were generated using the pT7GFP, pT7-MBP-GFP, and pT7-Trx-GFP vectors from our previous suite as templates (Correa et al., 2014). Vectors pMT/BiP/V5-his and pCDNA3.1 (Thermo Fisher Scientific) were used as templates for the generation of *Drosophila* and mammalian expression vectors, respectively. All cloning steps were made by RF-cloning (Unger et al., 2010). All the PCR amplifications of the different fragments for megaprimer generation as well as the RF reactions for cloning were performed as previously described (Correa et al., 2014). Selection of positive clones was performed by colony PCR by using Taq polymerase (Invitrogen^TM^) with the same primers used for megaprimer generation. PCR reaction was carried out as follows, 95°C for 3 min, 25 cycles of 95°C for 30 s, 67°C for 30 s, and 72°C for 2 min followed by a final extension step at 72°C for 5 min. Selected colonies were confirmed by sequencing. For the generation of the vector pT7GST, the GST gene derived from *Schistosoma japonicum* was amplified from the vector pGEX-4T-1 (GE Healthcare) with the primers T7GSTFor and GSTRev (**Table 1**) and cloned into pT7-Trx-GFP substituting the Trx moiety with GST gene. For the generation of the pT7pelB vector, the pelB sequence was amplified from the vector pET22b (Novagen) with the primers pelBT7For and pelBRev (**Table 1**) and cloned into the vector pT7GFP by RF cloning to generate the construct pT7pelB. This construct was then used as template with the primers T7 promoter and pelBinsRev and the generated megaprimer used for the insertion of the pelB signal sequence into vectors containing the T7 promoter and the fusion protein GST (pT7GST) or MBP (pT7MBP). The obtained vectors were named as pT7pelB-GST and pT7pelB-MBP, respectively, both containing the GFP gene sequence at the insertion site. For the generation of pDroEx, first we inserted the generic module [that includes HisTag, tobacco etch virus (TEV) recognition site, GFP gene, and StrepTagII] into pMT/BiP/V5-his (Invitrogen^TM^). We amplified from pT7GFP the generic module with the primers DrosModFor and DrosModRev (**Table 1**) and inserted in the pMT vector generating the construct pDroExHis. Then we substituted the HisTag with the Twin-Strep-tag^®^. To this aim, we first generated the Twin-Strep-tag^®^ by overlapping PCR with the primers TwinFor, TwinMedFor, and TwinRev (**Table 1**). This was then used as a megaprimer over pDroExHis to substitute the HisTag with the Twin-Strep-tag^®^ by RF-cloning resulting in the pDroEx construct. For cytoplasmic expression, the BiP signal sequence was removed from pDroEx. We first amplified the generic module containing the Twin-strep-tag^®^ from pDroEx with the primers TwinIntraFor and DrosModRev and used the generated megaprimer over the same vector resulting in the elimination of the signal sequence and generation of the construct pDroIn. The vector for cytoplasmic mammalian expression was developed by inserting our generic module between the cytomegalovirus (CMV) promoter and the BGH polyadenylation sequence of pCDNA3.1 vector (Invitrogen^TM^). In this regard, our generic module was amplified from pT7GFP with the primers CMVModFor and CMVModRev (**Table 1**) and used in an RF reaction with pCDNA3.1 as template generating the pCMVIn vector. For secretion to culture media, a signal peptide derived from the murine immunoglobulin kappa light chain was amplified from the vector pSV2-κ (Oppezzo et al., 2000) with the primers KappaFor and KappaRev (**Table 1**). The generated megaprimer was inserted into pCMVIn generating the construct pCMVExHis. To generate a vector for the expression and secretion of Fc fusion proteins in mammalian cells, we amplified the human IgG1 Fc fragment from the vector pFUSE-hIgG1-Fc1 (InvivoGen) with the primers Fc1For and FcRev. Five nanograms of the amplified fragment were used in a second PCR with primers Fc2For and FcRev (**Table 1**) to extend the annealing region at the 5′ with the destination vector. By this a megaprimer coding for a small linker (Gly-Ser), 3C protease recognition sequence and IgG1-Fc fragment was generated and used in a RF reaction with pCMVExHis to generate the vector pCMVExFc. The sequence of the entire vector suite was deposited in GenBank database and the codes for each vector are indicated in Supplementary Table 1. For the cloning of ROR-ED, the extracellular domain was amplified from cDNA derived from human lung carcinoma A549 cell line (ATCC^®^) by using the oligos ROR1For and ROR1Rev (**Table 1**). RF-cloning was used for the insertion of the ROR-ED megaprimer into pCMVExFc. In the case of ULBP2, primers ULBP2For and ULBP2Rev were used for megaprimer generation and the obtained product cloned into pCMVExFc in the same manner as for ROR-ED.

**Table 1:** List of oligonucleotides used for vector generation and cloning.

Primer name	Sequence 5′–3′	Characteristics	Vector name	Expression host	Localization
pelBT7For	GAAATAATTTTGTTTAACTTTAAGAAGGAGATATA CATATGAAATACCTGCTGCCGACCG	Insertion of pelB sequence	pT7pelB	*E. coli*	Periplasmic space
pelBRev	CCGCTACCGTGATGGTGATGGTGATGCGACAT GGCCATCGCCGGCTGGGC				
T7 promoter	TAATACGACTCACTATAGGG	Insertion of pelB sequence for fusion proteins	pT7pelB-Fusion	*E. coli*	Periplasmic space
pelBinsRev	CCCGAAGATCCGTGATGGTGATG				
T7GSTFor	CATCACCATCACCATCACGGATCTTCGGGAAT GTCTCCGATCCTGGGTTACTG	Insertion of GST gene	pT7GST	*E. coli*	Cytoplasmic space
GSTRev	CTGAAAATACAGGTTTTCCGATCCGCTACCGTCG GTAACGATGAATTCACG				
DrosModFor	GCCTTTGTTGGCCTCTCGCTCGGGAGATCTCAT CACCATCACCATCACGGTAGC	Insertion of generic module into pMT	pDroExHis	*Drosophila*	Secretion
DrosModRev	GTCGAGGCTGATCAGCGGGTTTAAACTCATTAC TTTTCGAACTGCGGGTGGC				
TwinFor	CCTTTGTTGGCCTCTCGCTCGGGAGATCTGGTTG GAGTCATCCACAATTCGAG	Substitution of HisTag with twin-Strep-tag	pDroEx	*Drosophila*	Secretion
TwinMedFor	GGAGTCATCCACAATTCGAGAAAGGCGGCGGCTC CGGAGGTGGATCAGGAGGTGGTTCCTGGTC ACACCCTCAATTCG				
TwinRev	CCCTGAAAATACAGGTTTTCCGATCCGCTCTTCTC GAATTGAGGGTGTGACC				
TwinIntraFor	CTAAAGGGGGGATCCGATCTCAATATGGCCTCT GGTTGGAGTCATCCACAATTCGAG	Elimination of BiP signal sequence	pDroIn	*Drosophila*	Cytoplasmic space
CMVModFor	GCTTGGTACCGAGCTCGGATCCACTAGTGCCACC ATGGGAGGATCGCATCACCATCACC	Insertion of the generic module into pCDNA3.1	pCMVIn	Mammalian cells	Cytoplasmic space
CMVModRev	GATCAGCGGGTTTAAACGGGCCCTCTAGACTCATT ACTTTTCGAACTGCGGGTGGC				
KappaFor	CTCACTATAGGGAGACCCAAGCTGGCTAGCCAC CATGGTATCCACACCTCAGTTCCTTG	Insertion of the kappa leader sequence	pCMVExHis	Mammalian cells	Secretion
KappaRev	GCTACCGTGATGGTGATGGTGATGCGATCCGATGT CACCTCTGGAGGCTGGAAAAATAG				
Fc1For	CCCGCAGTTCGAAAAGGGCAGCCTGGAAGTTCTGT TCCAGGGGCCCGACAAAACTCACACATGCCCAC	Insertionof Fc fragment	pCMVExFc	Mammalian cells	Secretion
Fc2For	GGATGAGCTCTACAAAAAGCTTGGATCCGGCAGCTGG AGCCACCCGCAGTTCGAAAAGGGCAGC				
FcRev	GGCACAGTCGAGGCTGATCAGCGGGTTTAAACTCAT TTACCCGGAGACAGGGAG				
ROR1For	GGATCGGAAAACCTGTATTTTCAGGGATCCCAAGAA ACAGAGCTGTCAGTCAGTGC	Amplification of ROR-ED	pCMVExFc	Mammalian cells	Secretion
ROR1Rev	GAACTGCGGGTGGCTCCAGCTGCCGGATCCGTACA GGATTTCCATTTTATTCTTCTCC				
ULBP2For	GGATCGGAAAACCTGTATTTTCAGGGATCCGGGCGA GCCGACCCTCAC	Amplification of ULBP2	pCMVExFc	Mammalian cells	Secretion
ULBP2Rev	GAACTGCGGGTGGCTCCAGCTGCCGGATCCCCTGAG TTGGGTTGTGCCTGAGG				

### Expression and Purification of pT7GST-GFP

BL21(DE3)pLysS *E. coli* cells were transformed with the pT7GST-GFP vector and growth overnight. Five milliliters of preculture were used to inoculate 500 ml of 2YT medium and when OD_600_ reached 0.8–1.0, induction was performed with 1 mM isopropyl β-D-1-thiogalactopyranoside (IPTG) for 4 h at 37°C. Cells were centrifuged and resuspended in PBS supplemented with protease inhibitor EDTA-free (Roche) and 0.5 mg/ml lysozyme (Sigma) (lysis buffer) and frozen at −80°C. Cells were thawed and lysed by sonication. Cell debris was removed by centrifugation at 18,000 × *g*, at 4°C for 25 min. Supernatant was passed through a 0.45 μm filter, and applied to a 1 ml GSTrap FF (GE Healthcare) column equilibrated in PBS. Column was washed with 10 column volumes (CVs) of PBS and recombinant protein eluted using 5 CVs of 50 mM Tris–HCl, pH 8.0 containing 10 mM reduced glutathione. The His-GST-GFP protein was then incubated with His-TEV protease (van den Berg et al., 2006) (1:10 w/w enzyme:protein ratio) at room temperature (RT) for 1 h and protein mixture was purified by IMAC by using a 1 ml HisTrap column (GE Healthcare) equilibrated in PBS. The cleaved target protein (GFP) was collected in the flow-through while the His-GST and the protease were eluted with a step gradient of elution buffer (50 mM NaPO_4_; 300 mM NaCl; 500 mM imidazole, pH 7.4). The purity of eluted proteins was analyzed by SDS-PAGE.

### Periplasmic and Cytoplasmic GFP Expression in *E. coli*

In order to express GFP in the periplasm or cytoplasm of *E. coli*, BL21(DE3) cells were transformed with pT7pelB, pT7pelB-GST, pT7pelBMBP, or pT7GFP vectors and growth overnight. One hundred milliliters of 2YT medium were inoculated with 1 ml of overnight culture and when OD_600_ reached 0.6–0.8, induction was performed with 0.25 mM IPTG for 16 h at 20°C. After induction cells were centrifugated at 4000 × *g* for 15 min and resuspended in PBS buffer. Cells were fixed for 5 min with 4% buffered formaldehyde (from paraformaldehyde), washed three times with PBS, and attached onto a poly-L-lysine coated slide and mounted in 70% glycerol pH 8.8. Specimens were imaged with an Olympus IX81 fluorescence microscope with a 100× immersion objective (NA = 1.4), and the images were deconvolved with Huygens Essential 4.5 (Scientific Volume Imaging B.V., Hilversum, Netherlands).

### Expression and Purification of GFP in *Drosophila* Cells

*Drosophila* S2 cells were grown at 28°C in Schneider’s *Drosophila* medium plus 10% fetal bovine serum (FBS) (Schneider’s complete medium) and co-transfected with pDroIn or pDroEx in conjunction with the resistance plasmid pCoPURO using Effectene^®^ Transfection Reagent (Qiagen). Transfection solution was prepared according to the manufacturer’s instructions. Both plasmids (pDroIn or pDroEx) at a ratio of 20:1 with pCoPURO were diluted up to 150 μl with Effectene buffer and mixed. Ten microliters of Enhancer was added, mixed, and incubated for 5 min at RT. Then 20 μl of Effectene Transfection Reagent was added and the mix incubated 15 min at RT. A T-25 flask containing 5 × 10^6^ cells incubated from the day before was used for transfection. Cells were washed with Schneider’s complete medium, resuspended with 4 ml of same medium and returned to the flask. Transfection solution was mixed with 1 ml of Schneider’s complete medium and added drop by drop to the cells. After 24 h of incubation at 28°C, the medium was replaced with 4 ml Schneider’s complete medium plus 1 ml conditioned medium. After 4 days incubation, puromycin at 6 μg/ml was added to the same medium for selection. Puromycin resistant cells were selected and the stable polyclonal population was adapted to serum-free medium (insect-XPRESS, Lonza). GFP expression was induced for 7 days with 5 μM CdCl2. Intracellular expression of GFP was visualized by confocal microscopy. Briefly, 100 μl culture containing cells were fixed for 10 min with 4% buffered formaldehyde (from paraformaldehyde), washed three times with PBS, and attached onto a poly-L-lysine coated slides and mounted in 70% glycerol pH 8.8. Specimens were imaged with a Zeiss LSM800 confocal microscope, and the images were deconvolved with Huygens Essential 4.5 (Scientific Volume Imaging B.V., Hilversum, Netherlands).

For secreted GFP, the cell culture was centrifugated at 6000 × *g* for 30 min and supernatant filtered and equilibrated to pH 8.0. Biotin blocking solution was added to block-free biotin from media (Biolock, iba) and the supernatant was injected to a 5 ml Strep-Tactin^®^XT Superflow^®^ column (iba) equilibrated with binding buffer (100 mM Tris/HCl pH 8.0, 150 mM NaCl, and 1 mM EDTA). Elution was performed in a single-step with 50 mM biotin in binding buffer and column was regenerated according to the manufacturer’s instructions.

### Expression of GFP in Mammalian Cells

HEK293T cells were grown in DMEM culture medium adapted to 5% FBS in six-well plates, and cells were seeded onto sterile poly-L-lysine coated cover slips and allowed to attach and expand. Then cells were transfected with pCMVIn and pCMVExFc vectors, using polyethylenimine (Polyethylenimine, linear, MW 25,000 Transfection Reagent Cat #23966; Polysciences, Inc.). Transfection begins by mixing a PEI solution with OPTIMEM (Thermo Fisher Scientific) to a final concentration of 20 μg/well. Each plasmid was then added to a separate polystyrol tube of Optimem to a final concentration of 2 μg/well. The PEI and DNA (10:1 μg/μg ratio) solutions were combined at RT for 30 min. After incubation, the transfection solution was added to the wells. Cells transfected with PEI alone were used as control. The medium was changed 24 h after transfection and cells were maintained for 3 days. The HEK293T transfected cells were fixed for 10 min with 4% buffered formaldehyde (from paraformaldehyde), washed three times with PBS, and attached onto a poly-L-lysine coated slides, and mounted in 70% glycerol pH 8.8. Specimens were imaged and images were deconvolved in the same manner as for *Drosophila* cells.

### Expression and Purification of ROR-ED and ULBP2

ROR1-ED was transfected into HEK293T cells as described above, but with a PEI concentration of 200 and 20 μg of plasmid per 150T Flask. Culture supernatants were harvested everyday and medium was replaced. Depending on the cell growth, production can be maintained up to 2 weeks. The supernatant was adjusted with the same volume of PBS buffer. The sample was filtered through a 0.22 μm filter immediately before being applied to a 1 ml HiTrap Protein A HP column (GE) equilibrated in binding buffer (20 mM phosphate buffer pH 7.4). Column was washed with 10 CVs of binding buffer, and the elution of FcROR1-ED was performed in two steps: first by applying 0.1 M sodium citrate pH 4.5 to remove bovine IgG from medium serum and then with 0.1 M sodium citrate pH 3.0 to elute Fc-ROR1-ED. The protein was dialyzed overnight in 50 mM HEPES, 500 mM NaCl, 150 mM KCl, 300 mM arginine, and 5% glycerol. After dialysis, FcROR1-ED was cleaved with 3C protease in a ratio 1:2 enzyme:target at 4°C overnight. The cleaved protein was subjected to a size-exclusion chromatography column on a Superdex 200 10/300 (GE Healthcare) equilibrated in the same buffer for further purification and characterization. Similar approach was applied for ULBP2 expression. After Protein A purification, ULBP2 protein was subjected to a size-exclusion chromatography column on a Superdex 200 10/300 (GE Healthcare) equilibrated in PBS buffer for characterization and further purification.

## Results

### Generation of the Extended Vector Suite

**FIGURE 1:**
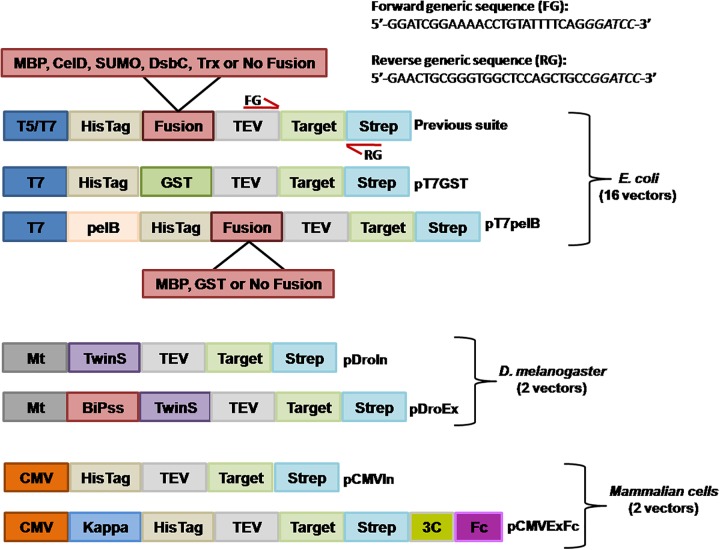
Schematic representation of the generated expression vectors. The different features of vectors are depicted. T5/T7, T5, or T7 promoter; fusion, corresponds to one of the different solubility enhancer-proteins or no fusion. Annealing regions for the forward generic sequence (FG) and reverse generic sequence (RG) are indicated with red arrows. Mt, metallothionein promoter; CMV, human cytomegalovirus immediate-early promoter/enhancer; pelB, pelB signal sequence; BiPss, BiP signal sequence; Kappa, murine Ig-κ leader sequence; TwinS, Twin-Strep-tag^®^; TEV, tobacco etch virus protease recognition site; Strep, *Strep*-Tag^®^II; 3C, human rhinovirus 3C protease recognition site; Fc, human IgG1 Fc fragment. All vectors contain the GFP gene at the insertion site that is replaced with the target gene by RF-cloning. The generic sequences to include at the 5′ of each primer are indicated at the top of the figure where italic letters correspond to a *Bam*HI site.

We routinely use our previously developed vector suite for the expression and purification of recombinant proteins with success (Correa et al., 2014), but some target proteins require native post-translational modifications, higher expression yields, and/or improved purity. Still, alternatives are available for the aforementioned requirements so we decided to extend our vector suite to overcome such limitations. These new vectors include the possibility to evaluate recombinant protein expression in the *E. coli* cytoplasm or periplasm by introducing the pelB signal sequence (pT7pelB, pT7pelB-GST, and pT7pelB-MBP), as well as cytoplasmic or secreted expression in eukaryotic hosts such as *Drosophila melanogaster* or mammalian cells (**Figure 1**). In addition, alternative purification strategies can be evaluated where for proteins expressed in *E. coli*, two purification methods are possible since target protein will contain an N-terminal HisTag and if no stop codon is included, a C-terminal *Strep*-Tag^®^II. Also two of our previous vectors include fusion with MBP. In this regard, target proteins can be efficiently purified by using amylose resins (Pattenden and Thomas, 2008). To further extend purification options in *E. coli*, we included an N-terminal fusion with GST to be used as a rapid purification method mainly for targets expressed at low yields (pT7GST). For recombinant protein expression in *Drosophila* cells, a Twin-Strep-tag^®^ was inserted at the N-terminal of the target gene for both vectors (pDroIn and pDroEx; for intracellular or secreted expression, respectively). In these cases, the sequence for the C-terminal *Strep*-Tag^®^II was maintained to conserve the generic reverse sequence. The introduction of two tags that bind to the same resin can be avoided in this case by including a stop codon at the end of the target, thus removing the *Strep*-Tag^®^II from the expressed protein. Two vectors for recombinant protein expression in mammalian cells were also developed for cytoplasmic expression (pCMVIn), or secretion into culture media (pCMVExFc) (**Figure 1**). For pCMVIn purification of target protein can be achieved by either an N-terminal HisTag or a C-terminal *Strep*-Tag^®^II. In the case of pCMVExFc, a signal peptide derived from the murine immunoglobulin kappa light chain was introduced for protein secretion as well as a C-terminal fusion with the human IgG1 Fc-fragment to allow purification of secreted proteins by Protein A affinity resins. The Fc portion can be then removed from the target protein by cleavage with the 3C protease.

All the generated vectors contain the same features than our previous series including, ampicillin resistance for selection in *E. coli*, the GFP gene at the insertion site, TEV cleavage recognition site at the N-terminal and a C-terminal *Strep*-Tag^®^II. The nucleotide sequences flanking the insertion site are maintained in all vectors (**Figure 1**), so the same megaprimer can be used to replace the GFP gene with the target DNA by RF-cloning through the entire suite. The 20 different vectors of the extended suite allows the evaluation of different promoters, solubility enhancing proteins, purification strategies, host and host compartments involving 16 vectors for expression in *E. coli*, 2 for expression in *Drosophila*, and 2 for expression in mammalian cells (**Figure 1**).

### Rapid and Effective Purification Protocol of GST-Fusion Proteins

Despite IMAC purification is a very robust, fast, and efficient method, it has been observed that for low expressing targets, recombinant proteins are eluted with several contaminants derived from the host (Bolanos-Garcia and Davies, 2006). In this regard, other tags such as *Strep*-Tag^®^II showed better specificity with similar beneficial properties that the ones found with the HisTag (Lichty et al., 2005), so it was included in our vectors as a C-terminal fusion.

**FIGURE 2:**
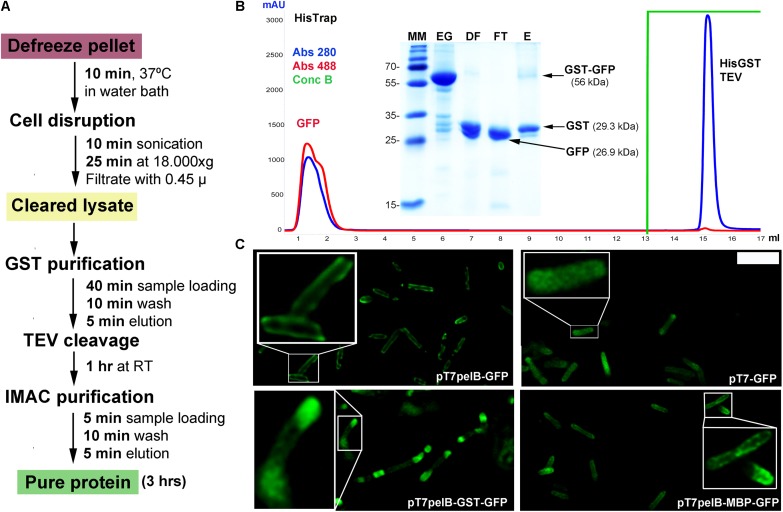
GFP expression in *E. coli*. **(A)** Purification workflow of GST fusion proteins. **(B)** IMAC purification of the cleaved GST-GFP protein. Absorbance was measured at 280 nm (blue line) and 488 nm (red line). Purified fractions were loaded into a 15% SDS-PAGE for visualization and assessment of protein purity. MM, molecular marker; EG, eluted fraction from GST purification; DF, digested fraction with TEV protease; FT, flow-through from IMAC purification; E, eluted fraction from IMAC purification. The expected molecular weight for the different proteins is indicated in parenthesis and the molecular mass of the marker indicated in kilodalton at the left of the gel. **(C)** Deconvolved widefield microscopy of periplasmic GFP expression (pT7pelB-GFP, pT7pelB-GST-GFP, and pT7pelB-MBP-GFP) or cytoplasmic GFP expression (pT7-GFP) in *E. coli*. A 2.5× zoom of selected cells is indicated with white boxes to illustrate expression differences. Scale bar: 3 μm.

We still wanted to incorporate an additional purification method for proteins expressed in *E. coli*, and generated a vector for fusion with GST. In this regard, GST demonstrated to be a very effective purification tag with which the target protein can be obtained in an almost pure state in a single-step (Harper and Speicher, 2011). By using the vector pT7GST, a rapid and efficient purification method was designed were the fusion protein can be purified from the cell pellet; the His-GST tag cleaved, removed, and target protein obtained after only 3 h (**Figures 2A,B**). We transformed BL21(DE3)pLysS cells with pT7GST-GFP and performed an expression test. After induction, cells were centrifuged, resuspended in lysis buffer, and frozen. Following thawing, the cells were disrupted and the cleared lysate injected into a GSTrap column. The His-GST-GFP protein was eluted with reduced glutathione and incubated with His-TEV protease at 25°C for 1 h and the protein mixture was injected into a HisTrap column (**Figure 2B**). Absorbance was measured at 280 and 488 nm. The later was included since it corresponds to the absorbance peak of GFP. As it can be observed in the chromatogram, the GFP portion of the fusion is obtained in the flow-through (red line, 488 nm) while the GST portion and TEV protease are obtained after applying 100% buffer B (containing 500 mM imidazole). These results were confirmed by SDS-PAGE revealing that an almost pure target protein was obtained with this protocol (**Figure 2B**).

### Multi-Compartment Expression of GFP in *E. coli*

Several strategies have been developed to allow the proper formation of disulfide bonds in the cytoplasm of *E. coli* (Hatahet et al., 2010; Nguyen et al., 2011; Lobstein et al., 2012; Nozach et al., 2013). However, the production of many target proteins requiring a native disulfide bond pattern still fails when produced in the *E. coli* cytoplasm. In this sense, periplasmic translocation by the use of the pelB signal sequence can be a valuable alternative to consider favoring the production of recombinant proteins with a native disulfide bond pattern (Sockolosky and Szoka, 2013). The pelB signal sequence was inserted into the vector pT7-GFP upstream the HisTag to obtain the vector pT7-pelB-GFP, to direct the target protein into the periplasmic space (**Figure 1**). *E. coli* cells were transformed with pT7-GFP or pT7pelB-GFP for comparison, and the expression of GFP was induced. The sub-cellular localization of GFP was determined by fluorescence microscopy, where it was revealed that while for pT7-GFP the fluorescence was observed homogeneously along the entire cell, for pT7pelB-GFP, fluorescence was concentrated at the periphery of the cell, in accordance with a periplasmic localization (**Figure 2C**). In addition, vectors for periplasmic localization of target proteins as a fusion with the solubility enhancer-proteins GST and MBP were generated (pT7pelB-GST and pT7pelB-MBP, respectively). As depicted in **Figure 2C**, similar results were obtained for both constructs.

Hence in regard with recombinant protein expression in *E. coli*, besides the possibility to evaluate different promoters and solubility enhancer proteins, an efficient and rapid purification protocol can be performed by fusion with GST as well as the possibility to direct target proteins to the periplasmic space to allow the proper formation of disulfide bonds.

### Multi-Compartment Expression of GFP in *Drosophila* Cells

**FIGURE 3:**
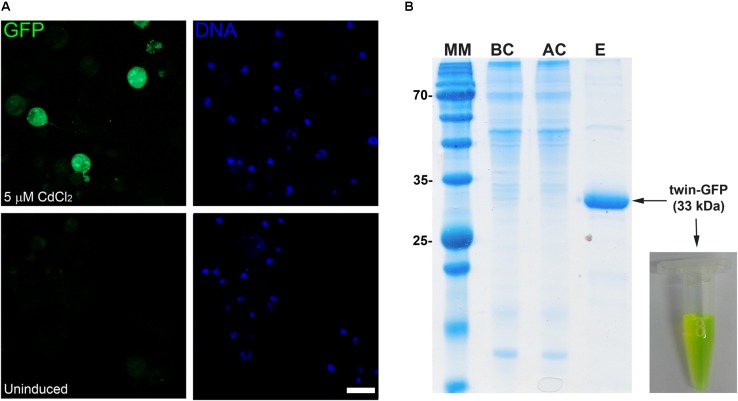
Multi-compartment expression of GFP in *Drosophila* cells. **(A)** Confocal microscopy of cytoplasmic GFP expression in *Drosophila* S2 cells. A strong GFP signal is observed in the cytoplasm of several CdCl_2_-induced cells (green) while it is absent in uninduced cells. DNA was stained with methyl green (blue) (Prieto et al., 2014). Scale bar: 15 μm. **(B)** Fifteen percent SDS-PAGE of GFP purification from *Drosophila* culture media with a Strep-TactinXT resin. MM, molecular marker; BC, before column; AC, after column; E, eluted fraction with 50 mM biotin. The purified GFP protein containing the N-terminal twin-Strep-tag (twin-GFP) is indicated with an arrow with the expected molecular mass in parenthesis. Numbers at left of gel corresponds to molecular mass of the marker in kilodalton. A photograph of the eluted twin-GFP protein is shown.

In order to increase the versatility of the vector suite, some plasmids were developed for expression in other hosts such as *Drosophila* cells. In this regard, we generated two vectors one for cytoplasmic expression named pDroIn, and other for secretion to the culture media via the BiP signal sequence named pDroEx (**Figure 1**). To test both vectors, GFP was expressed in *Drosophila* S2 cells in conjunction with the pCoPuro selection vector. The cytoplasmic expression of GFP with pDroIn was confirmed by confocal microscopy, where strong fluorescence was observed for cells induced for 7 days with 5 μM CdCl_2_ while it was absent in uninduced cells, reveling a tight regulation of the target gene (**Figure 3A**). For the secretion construct, after induction recombinant protein was purified from the culture media by using a Strep-Tactin^®^XT column that tightly binds to the Twin-Strep-tag^®^ (Yeliseev et al., 2017). As shown in **Figure 3B**, GFP protein was purified with high purity in a single-step from the culture media by using this approach obtaining a final yield of 12 mg/l. In addition, a strong green color was visible in the eluted fraction in accordance with a properly folded protein (**Figure 3B**).

### Multi-Compartment Expression of Recombinant Proteins in Mammalian Cells

**FIGURE 4:**
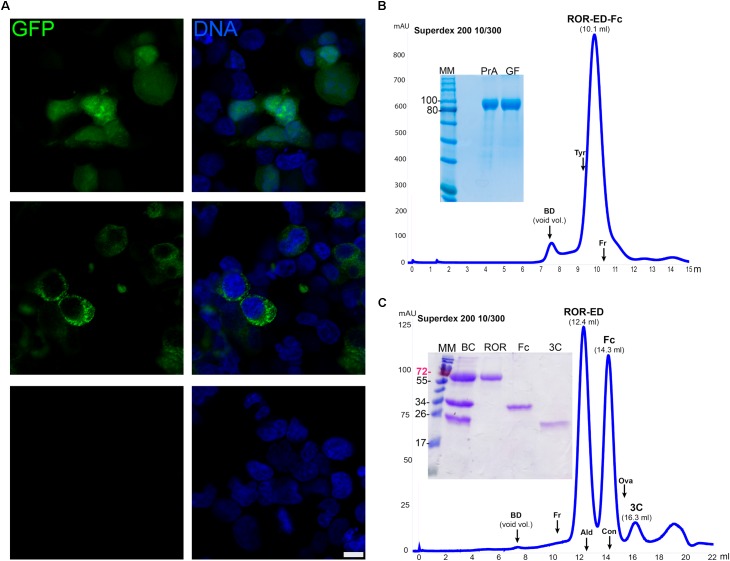
Recombinant protein expression in mammalian cells. **(A)** Confocal microscopy of HEK293T cells transfected with pCMVIn-GFP (upper panels), pCMVEx-GFP-Fc (middle panels), or no vector (Mock, lower panels). GFP fluorescence was detected for the transfected cells (green) with both plasmids either homogeneously in the cytosol (pCMVIn-GFP), or with a cytoplasmic punctate pattern (pCMVEx-GFP-Fc), whereas no GFP signal was detected in Mock transfected cells. DNA was counterstained with methyl green (Prieto et al., 2014) (blue). Scale bar: 10 μm. **(B)** Purification and characterization of secreted ROR-ED-Fc. Fusion protein was purified by Protein A and gel filtration on a Superdex 200 10/300 giving a mayor peak at 10.1 ml corresponding to the pure protein as shown in the 12% SDS-PAGE at left. MM, molecular marker; PrA, eluted fraction from Protein A purification; GF, peak indicated as ROR-ED-Fc on the gel filtration chromatogram. **(C)** Fusion protein was cleaved with 3C protease and injected into a Superdex 200 10/300. ROR-ED eluted as a pure and homogeneous protein. Elution volume for each protein is indicated over the peaks in parenthesis. A 15% SDS-PAGE was loaded with fractions from each peak to corroborate purity. BC, before column; ROR, peak at 12.4 ml; Fc, peak at 14.3 ml; 3C, peak at 16.3 ml. Elution volume of different standards are indicated by an arrow over the chromatograms, were BD accounts for Blue Dextran (void volume); Tyr, Thyroglobulin (669 kDa); Fr, Ferritin (440 kDa); Ald, Aldolase (158 kDa); Con, Conalbumin (75 kDa); Ova, Ovalbumin (43 kDa). Numbers at left of gels corresponds to molecular mass of the marker in kilodalton.

For some targets, specific features like the presence of biological partners or a defined glycosylation pattern may be fundamental for the expression of a functional protein. This is particularly important for the case of several human therapeutic proteins, where the use of human cell lines as the expression host may be a better alternative (Swiech et al., 2012; Dumont et al., 2016). In this context, we developed for mammalian cells two vectors, one for cytosolic expression (pCMVIn) and other for secretion into culture media (pCMVExFc). GFP was transiently expressed in the cytoplasm of HEK293T cells by using the vector pCMVIn that already contains the GFP gene. This was confirmed by confocal microscopy where a strong and homogeneous cytosolic fluorescence was detected in transfected cells while this was absent from Mock transfected cells (**Figure 4A**). Similar results were observed for cells transfected with the plasmid for secretion and fusion with the Fc fragment pCMVExFc, where strong cytoplasmic GFP signal was detected with a punctate pattern, in accordance with the vesicular compartment of the secretion route (**Figure 4A**).

To test these vectors even further, we expressed two challenging proteins such as ROR1-ED and ULBP2, which contain several N-glycosylation sites and disulfide bonds. The gene for ROR1-ED was cloned into the vector pCMVExFc to generate the construct pCMVEx-ROR-Fc. HEK293T cells were transfected, culture changed each day for a period of 14 days and ROR-ED-Fc fusion protein was purified from culture media by Protein A. From previous studies, it was shown that the N-glycosylation on ROR1 is responsible for an increase of around 25 kDa in the electrophoretic mobility (Kaucká et al., 2011). Our ROR-ED-Fc construct has a predicted molecular weight of 72 kDa, however, our purified protein migrates as a 100 kDa protein on an SDS-PAGE (**Figure 4B**). This is in accordance with the reported increase in the electrophoretic migration due to the incorporation of the N-glycans.

To determine the oligomeric state of the fusion protein, analytical size exclusion chromatography (ASEC) was performed revealing a homogeneous protein with an estimated molecular weight corresponding to a tetrameric state if we consider the incorporated glycans (**Figure 4B**). In addition, as shown in the SDS-PAGE in **Figure 4B** a pure protein was obtained after using two purification steps (Protein A and size exclusion chromatography) with a final yield of 1.1 mg/l. Similar results were obtained for ULBP2 where fusion protein was purified to homogeneity after Protein A purification and ASEC, with a final yield of 3 mg/l (Supplementary Figure S1).

To confirm that ROR-ED remains soluble and homogeneous after tag removal, we proceeded to the cleavage and purification of the fusion protein. For this purpose, we purified in a first step the secreted protein from culture media by Protein A. The eluted fraction was dialyzed for buffer exchange and incubated with 3C protease at 4°C overnight. Sample was filtered and injected into a gel filtration column to separate the Fc fragment and 3C protease from ROR-ED. As shown in **Figure 4C**, the cleavage was successful and ROR-ED remained soluble and homogeneous after tag removal. Moreover, size exclusion chromatography was very effective in the separation of ROR-ED, Fc fragment and 3C protease as seen from the chromatogram and the SDS-PAGE (**Figure 4C**). The cleaved protein still contains a HisTag and a *Strep*-Tag^®^II, so they can be used to separate target proteins that due to their molecular weight, cannot be separated from Fc fragment or 3C protease by gel filtration. Again, the predicted molecular weight for the cleaved protein is 46 kDa, however, our purified and cleaved protein migrates as a 70 kDa protein in an SDS-PAGE, in accordance with the reported incorporation of N-glycans (**Figure 4C**). A similar scenario occurs with the Fc fragment that with an expected molecular mass of 25.7 kDa migrates at around 34 kDa due to a glycosylation site at the Asn297 (Higel et al., 2016). Taking this into account, the ASEC reveals a dimeric state for the cleaved ROR-ED as well as for the Fc fragment as expected. Finally, the identity of the purified proteins, ROR-ED and Fc fragment, were confirmed by peptide fragmentation using MALDI-MS/MS (Lima et al., 2011).

Altogether, these results show that by using the extended vector suite, recombinant protein expression can be evaluated in different hosts and cell compartments, in combination with distinct purification alternatives, which can be essential for the soluble expression of challenging proteins in sufficient amounts.

## Discussion

Recombinant protein expression does not always lead to obtaining a soluble, pure, and homogeneous product. Many target proteins can accumulate as insoluble aggregates known as inclusion bodies, or are expressed at very low yields. To circumvent these obstacles, several parameters that enhance protein stability and folding leading to the production of a functional protein can be evaluated (Vincentelli et al., 2011; Correa and Oppezzo, 2015). However, the evaluation of some factors including different promoters, distinct cellular localization or an alternative host, may require the cloning of the target gene into multiple vectors. In the present study, we have expanded our vector suite also for the evaluation of recombinant protein expression in different cell compartments as well as alternative hosts. By using RF-cloning, the same megaprimer can be now introduced in a parallel manner into the 20 vectors of our extended suite.

In addition to success in recombinant protein expression, the final yield will also depend on the employed purification method. For example, there are several native proteins from *E. coli* that can interact with metal-chelating resins including the ones used for IMAC, and are commonly eluted with the target. This is especially important when working with proteins that are expressed at low yields, making the purification process difficult and leading to product loss (Bolanos-Garcia and Davies, 2006). With this in mind, we extended the purification options and included a vector for fusion with GST under the control of the T7 promoter, pT7GST. The GST protein has been extensively used as a solubility-enhancer protein that also allows single-step purification by interacting with glutathione resins and elution is performed under mild conditions with reduced glutathione (Smith and Johnson, 1988; Harper and Speicher, 2011). With the generated pT7GST vector and if the stop codon of the target gene is avoided, three affinity tags will be available allowing purification of the target through the HisTag, GST fusion and/or *Strep*-Tag^®^II. By using fusion with GST, a rapid and effective purification method was designed were recombinant proteins can be obtained in an almost pure state from cell pellets within 3 h. This includes a capture step with a GST resin, the proteolytic cleavage of the fusion protein with TEV protease and the subsequent removal of GST portion and protease by IMAC. Impurities derived from different oligomeric states of the target protein can then be separated by size exclusion chromatography if necessary.

Many protein targets require the proper formation of disulfide bonds for correct folding, stability, and or activity. In this regard, several strategies were developed to allow the efficient formation of native disulfide bonds in the *E. coli* cytoplasm. These include the use of engineered *E. coli* mutant strains that contain a more oxidizing cytoplasm (Bessette et al., 1999), co-expression in these cells of the DsbC isomerase (Shuffle *E. coli* strain, NEB) (Lobstein et al., 2012), co-expression in the cytoplasm of a sulfhydryl oxidase, and a disulfide isomerase (Hatahet et al., 2010; Nguyen et al., 2011) or fusion of the target protein with DsbC (Nozach et al., 2013). Although these strategies permitted the correct expression of many challenging proteins, several targets still fail to form a native disulfide bond pattern and soluble expression is not achieved in the *E. coli* cytoplasm. In these cases, the translocation to a more oxidizing environment like the periplasmic space, may circumvent folding problems (Berkmen, 2012; Sockolosky and Szoka, 2013). An additional advantage of targeting to this compartment is that purification of proteins is usually easier, since it contains a less complex protein mixture than the cytoplasm (Mergulhão et al., 2005). Still, if the target gene is expressed at high rates, the translocation machinery of the bacteria can be saturated leading to toxic effects for the cell and reducing protein yields. Considering these issues, regulation of expression intensity may be an important factor. The latter can be achieved by using, for example, the Lemo21(DE3) (NEB) strain, were transcription rates from the T7 promoter can be finely tuned to avoid the saturation of the translocation machinery (Schlegel et al., 2013). To this aim, we generated three vectors containing the pelB signal sequence for recombinant protein expression under the control of a T7 promoter. As shown in this work, by using these vectors, proteins can be effectively translocated to the periplasmic space of *E. coli.* Target proteins cloned into these vectors will contain N-terminal HisTag, followed by a TEV cleavage site for tag removal and if the stop codon is avoided, a C-terminal *Strep*-Tag^®^II. Additionally by fusion with GST or MBP, yields can be increased and because these proteins are also purification tags, up to three different affinity purification options can be employed.

Recombinant protein expression is not limited to *E. coli*, making the use of eukaryotic hosts inevitable for the expression of many challenging proteins. In addition, depending on the required quantities and quality of the target protein, a single host cell line may be the optimal expression system while others will fail in the expression of the same target (Karste et al., 2017). To tackle this scenario, we extended our vector suite for expression of the target gene in different hosts including *D. melanogaster* and mammalian cell lines.

*Drosophila melanogaster* Schneider 2 insect cell line (S2) has emerged as an attractive host for recombinant protein expression by combining moderate cost and operational complexity with the formation of post-translational modifications including glycosylation and proper disulfide bond formation. Unlike other insect cells, S2 can be transfected independently of viral infection. Moreover, a large number of plasmid copies are integrated into the cellular genome and proteins can be effectively secreted to the media if a secretion signal peptide is included, facilitating downstream processing (Moraes et al., 2012). We generated our *D. melanogaster* vectors based on the commercial pMT/BiP/V5-his vector (Invitrogen^TM^) that use the metallothionein promoter (pMt) to allow an inducible expression with metals like CuSO_4_ and CdCl_2_. In addition, it contains the BiP signal sequence to secrete recombinant proteins into the culture media. Our generic module contains an N-terminal HisTag and a C-terminal *Strep*-Tag^®^II. However, in our hands, the expression of some His-tagged proteins in *D. melanogaster* resulted in poor yields. Moreover, if we use the C-terminal *Strep*-Tag^®^II for purification, 12 extra residues will remain in the purified protein. Thus, we decided to substitute the N-terminal HisTag with the Twin-Strep-tag^®^ for our *D. melanogaster* vectors, since it demonstrated great performance for purification of diluted proteins from culture media (Schmidt et al., 2013). With the presented format a highly pure and almost native protein could be obtained after tag removal with TEV protease where only two extra residues (Gly-Ser) will remain at the N-terminal of the target. In addition, we used these plasmids in combination with the selection vector pCoPURO, which confers resistance to puromycin. This system minimizes transfected S2 cell selection time where non-transfected cells are eliminated within 3 days (Iwaki et al., 2003; Moraes et al., 2012). Finally, GFP was successfully expressed and secreted to the culture media with the pDroEx vector, and a highly pure protein was obtained after a single purification step by combining the Twin-Strep-tag^®^ with Strep-Tactin^®^XT column.

Obtaining a soluble and homogeneous product may not be the only requirement, as for many therapeutic proteins like monoclonal antibodies, where the incorporation of a specific glycosylation pattern, like the one found in their natural host can have important effects in the proper function of the produced target (Swiech et al., 2012; Liu, 2015; Dumont et al., 2016; Vazquez-Lombardi et al., 2018). Such requirements make the use of human cell lines for recombinant therapeutic protein production an attractive option (Swiech et al., 2012; Dumont et al., 2016). With this purpose, we included vectors for constitutive expression in mammalian cell lines under the control of the human CMV immediate-early promoter/enhancer. We generated one vector for cytoplasmic expression and one for secretion into the culture media. The latter was achieved by including a signal peptide derived from the murine Ig-κ chain leader sequence, previously used in our laboratory for the successful expression and secretion of chimeric antibodies (Oppezzo et al., 2000). In addition, a C-terminal fusion with the antibody Fc fragment was also included to allow purification by Protein A. This last strategy has been extensively used for the purification of antibodies and Fc-fusion proteins secreted to culture media (Fisher et al., 2006; Flanagan et al., 2007). In this study, the cytoplasmic expression of GFP was successfully achieved with the vector pCMVIn as it was seen by confocal microscopy. Similar results were obtained with the vector pCMVExFc containing the GFP gene; in which GFP was detected inside the cells forming punctate patterns in accordance to be transported through the secretion route.

Finally, by using the pCMVExt-Fc vector, we expressed two challenging proteins, the human ROR1-ED that is involved in cancer progression of a number of blood and solid malignancies (Hojjat-Farsangi et al., 2014) and the ligand for the NKG2D activating receptor on the surface of NK cells, ULBP2 (Sutherland et al., 2006).

ROR1-ED contains several disulfide bonds and has multiple N-glycosylation sites that regulate localization and signaling (Kaucká et al., 2011; Hojjat-Farsangi et al., 2014). After purification from culture media with Protein A, cleavage with 3C protease for Fc removal and size exclusion chromatography a soluble, pure, and dimeric target protein was obtained. In the case of ULBP2, a soluble, pure, and homogeneous protein was also obtained as a fusion with Fc fragment. These results validate the vector and the employed purification strategy.

Although all the cloning steps in the present study were performed using RF-cloning methodology, this vector suite is adaptable to other cloning methods including recombination-assisted megaprimer (RAM-cloning) (Mathieu et al., 2014), circular polymerase extension cloning (CPEC) (Quan and Tian, 2011), In-Fusion (Zhu et al., 2007), QuickStep-cloning (Jajesniak and Wong, 2015), and exponential megapriming PCR (EMP-cloning) (Ulrich et al., 2012). These alternative methods are important to consider specially if working with long DNA fragments (>2.5 kb) where it has been observed that RF-cloning efficiency decreases considerably (Ulrich et al., 2012; Mathieu et al., 2014).

We believe that our extended vector suite will facilitate cloning steps and the evaluation of several expression conditions including different cell compartments, expression hosts, and purification strategies, essential for obtaining challenging proteins in a functional state.

## Author Contributions

CO, DP, PO, and AC conceived and designed the experiments. CO, DP, CA, and AC performed the experiments. CO, DP, CA, PO, and AC analyzed the data. CO, PO, and AC wrote the paper.

## Conflict of Interest Statement

The authors declare that the research was conducted in the absence of any commercial or financial relationships that could be construed as a potential conflict of interest.
