# Supplementary material for: Multi-Compartment and Multi-Host Vector Suite for Recombinant Protein Expression and Purification
Source: Front Microbiol. 2018 Jun 27;9:1384. doi: 10.3389/fmicb.2018.01384 (PMC6030378; doi:10.3389/fmicb.2018.01384)
Supplement: Supplementary file 2 [file Image_1.PDF]

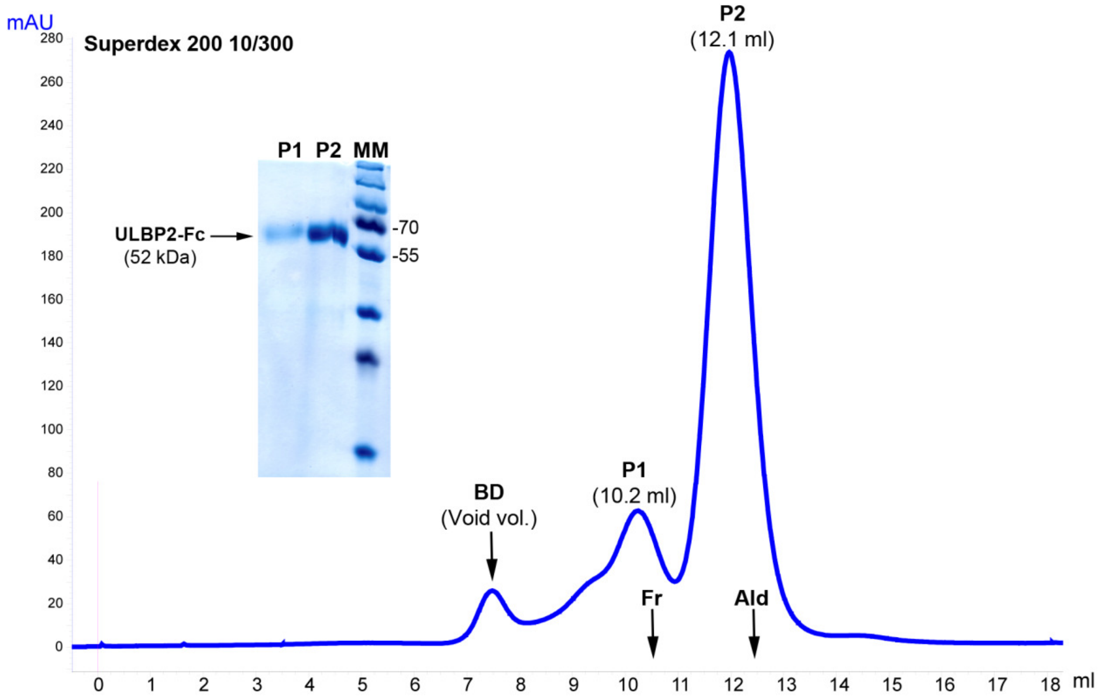

Supplementary Figure 1. Expression and purification of ULBP2-Fc in mammalian cells. HEK293T cells were transfected with pCMVEx-ULBP2-Fc, and recombinant protein was purified from the supernatant by protein A affinity chromatography. The eluted protein was injected into an ASEC column for further purification. As seen on the chromatogram ULBP2-Fc protein can be purified to homogeneity eluting as a mayor peak at 12.1 ml. A 12 % SDS-PAGE of ULBP2 purification with Superdex 200 10/300 column is shown, where a band with a slightly higher molecular mass than expected is observed. This last can be explained due to the presence of glycosylation sites present in ULBP2 protein and the Fc portion. P1, peak at 10.2 ml; P2 peak at 12.1 ml; MM, molecular marker. The purified ULBP2-Fc protein is indicated by an arrow with the expected molecular mass in parenthesis. Numbers at right of gel corresponds to the molecular mass of the marker in kDa. Elution volume of different standards are indicated by an arrow over the chromatogram, were **BD** accounts for Blue Dextran (void volume); **Fr**, Ferritin (440 kDa) and **Ald**, Aldolase (158 kDa).
